# Editorial: Radiotherapy for esophageal cancer: Molecular mechanism and novel targets to improve radiosensitivity

**DOI:** 10.3389/fonc.2023.1188010

**Published:** 2023-04-03

**Authors:** Yanqing Liu, Adam Yongxin Ye, Jordan Lu, Jiaxing Yang, Xiao Zheng, Xiaodong Li

**Affiliations:** ^1^ Herbert Irving Comprehensive Cancer Center, Columbia University, New York, NY, United States; ^2^ Howard Hughes Medical Institute, Boston Children’s Hospital, Harvard Medical School, Boston, MA, United States; ^3^ Department of Tumor Biological Treatment, The Third Affiliated Hospital of Soochow University, Changzhou, China; ^4^ Africa Hepatopancreatobiliary Cancer Consortium (AHPBCC), Mayo Clinic, Jacksonville, FL, United States; ^5^ Department of Oncology, Third Affiliated Hospital of Soochow University, Changzhou, China

**Keywords:** esophageal cancer, radiotherapy, chemotherapy, prognostic marker, overall survival

Esophageal cancer (EC) is the seventh most common cancer and the sixth leading cause of cancer-related deaths worldwide (1, 2). Multimodal treatment with curative intent, combined with surgery, chemotherapy, and/or radiotherapy, has been developed for patients with locally advanced EC (3). Radiotherapy is one of the main strategies for EC treatment. Radiotherapy aims to maximize damage to cancer cells, while minimizing harm to healthy cells. However, radiotherapy alone is not effective. A range of 70%-80% of patients have uncontrolled local tumor lesions, as well as tumor recurrence in the radiation target area. Resistance to radiation therapy is polymodal and associated with a number of biological alterations both within the tumor itself and in the surrounding microenvironment. Studies on key molecules in the signal pathways or immune escape process is expected to reveal the molecular mechanism of EC radio-resistance, and to provide new strategies for improving the radiosensitivity of cancer cells and reducing the rate of local recurrence.

In order to reflect the latest research advances in the field of EC radiotherapy, we organized this Research Topic entitled Radiotherapy for esophageal cancer: molecular mechanism and novel targets to improve radiosensitivity. We now have published 5 research articles in this topic. These papers focus on the mechanism and treatment of EC from different angles. Here we summarize their major findings.

ELAV like RNA binding protein 1 (ELAVL1, or HuR) is an oncogenic RNA binding protein in different cancer types (4, 5). Hu et al investigated whether knockdown of HuR could promote the anti-EC effect of X-ray. Compared with X-ray treatment only, downregulation of HuR plus X-ray has a much stronger effect on EC cell proliferation and apoptosis. This synergic effect was also validated in xenograft mouse model. Mechanistically, HuR binds the 3-UTR of Snail mRNA. This binding will stabilize Snail mRNA and elevate the level of Snail protein. These results suggested a combination method to strengthen the effect of EC radiotherapy. In another study by Huang et al, authors explored another combination therapy in EC: concurrent chemoradiotherapy (CRT) after induction chemotherapy (IC). In this clinical study, authors observed enhanced efficacy of the IC plus CRT treatment compared with CRT only. Both overall survival (OS) and progression-free survival (PFS) of EC patients in the combination group are longer than CRT group. Rochigneux et al. once carried out a monocentric phase II study (FIDUCOR-study, NCT02526134) to evaluate the fiducial markers (FMs) implantation on CRT in EC treatment. Significantly, they observed a 100% modification of the growth-tumor-volume-dimension. This means that FMs-implantation under endoscopic ultrasound is an applicable method in ES therapy. Identification of suitable and precise prognostic marker is beneficial to the treatment of EC patient. There is evidence that plasma fibrinogen and serum albumin level (FA score) can predict the survival time in certain cancer types. However, its application in esophageal squamous cell carcinoma (ESCC) remains elusive. In a retrospective study, Wang et al. analyzed 154 ESCC patients who underwent concurrent radiochemotherapy. They revealed that a higher pretreatment FA score was associated with poorer treatment effect and shorter median OS. This study gives valuable suggestion for the choice of treatment methods for ESCC in the future. There is still a question whether the pretreatment FA score has similar prognostic value in esophageal adenocarcinoma. Prognostic markers based on blood molecules or cells are also useful in other cancers, for example gastric cancer. The peripheral blood inflammatory index and nutritional index can be defined in different settings, such as the platelet lymphocyte ratio (PLR), neutrophil lymphocyte ratio (NLR), lymphocyte monocyte ratio (LMR), systemic inflammation response index (SIRI), pan-immune-inflammation value (PIV), systemic immune-inflammation index (SII), and prognostic nutrition index (PNI). Some of them may have great prognostic value in locally advanced gastric cancer (LAGC). Wang et al. assessed the efficacy of these indexes in LAGC prognosis and discovered that among them NLR was the best marker for predicting the survival in LAGC patients treated with adjuvant chemoradiotherapy after D2 dissection. A higher NLR was significantly related to a shorter OS and disease-free survival.

To conclude this editorial, we successfully compiled 5 excellent research papers in this topic. We wish these papers could ignite novel ideas in EC research and treatment, especially about its radiotherapy. We also expect to see more exciting developments in this field in the near future.

## Author contributions

YL, JL, and XL wrote the manuscript. AY, JY, and XZ reviewed and revised the manuscript. The authors read and approved the final manuscript. The requirements for authorship have been met. Each author believes that the manuscript represents honest work.
